# Supplementary material for: Establishing the Prevalence of Osteomalacia in Arab Adolescents Using Biochemical Markers of Bone Health
Source: Nutrients. 2022 Dec 16;14(24):5354. doi: 10.3390/nu14245354 (PMC9784134; doi:10.3390/nu14245354)
Supplement: Supplementary file 1 [file nutrients-14-05354-s001.zip › nutrients-2095999-supplementary.pdf]

Table S1. Age- and sex-specific normal ranges used for serum alkaline phosphatase (ALP) and inorganic phosphorous (Pi).

**Age groups (years)** ALP (U/l) [16] *	**Boys**	**Girls**
1-9	156-369	156-369
10-12	141-460	141-460
13-14	127-517	62-280
15-16	89-365	54-128
17-18	59-164	48-95
Pi (mmol/l) [17]		
5–13	1.19–1.74	1.29–1.68
14–15	1.13–1.52	1.13–1.58
16–17	1.0–1.52	1.0–1.52

*References ranges established from Abbott ARCHITECT analyser.

Table S2. Normality of Parameters Assessed.

**Parameters**	**Girls**		**Boys**	
N	Skewness	Kurtosis	Skewness	Kurtosis
Age (years)	0.3 ± 0.1	-0.9 ± 0.1	0.1 ± 0.1	-0.8 ± 0.1
Height (cm)	-0.1 ± 0.1	0.8 ± 0.1	-0.6 ± 0.1	0.2 ± 0.1
Weight (kg)	0.5 ± 0.1	0.0 ± 0.1	0.3 ± 0.1	0.5 ± 0.1
BMI (kg/m^2)	0.8 ± 0.1	1.4 ± 0.1	0.6 ± 0.1	0.9 ± 0.1
Ca (mmol/l)	-0.1 ± 0.1	2.4 ± 0.1	0.2 ± 0.1	3.1 ± 0.1
Pi (mmol/l)	0.3 ± 0.1	0.3 ± 0.1	0.5 ± 0.1	2.4 ± 0.1
ALP (U/l)	2.3 ± 0.1	7.6 ± 0.1	2.7 ± 0.1	9.7 ± 0.1
25(OH)D (nmol/l)	2.5 ± 0.1	8.7 ± 0.1	2.6 ± 0.1	16.7 ± 0.1

Note: For sample size >300, either an absolute skewness value ≤2 or an absolute kurtosis (excess) ≤4 are used as reference values for determining considerable normality [22, 23].
